# Electroacupuncture for oculomotor nerve palsy after chemotherapy: A case report

**DOI:** 10.1097/MD.0000000000038547

**Published:** 2024-06-14

**Authors:** Chun Du, Chao Lu, Guangliang Li, Dehou Deng, Weiji Chen

**Affiliations:** a Tianshui and Wulin Street Community Health Service Center of Gongshu District, Hangzhou, China; b Department of Traditional Chinese Medicine, Zhejiang Cancer Hospital, Hangzhou, China; c Department of Breast Medical Oncology, Zhejiang Cancer Hospital, Hangzhou, China; d Department of Acupuncture and Moxibustion, The Third Affiliated Hospital of Zhejiang Chinese Medical University (Zhongshan Hospital of Zhejiang Province), Hangzhou, China.

**Keywords:** breast cancer, case report, chemotherapy, electroacupuncture (EA), oculomotor nerve palsy (ONP)

## Abstract

**Introduction:**

Oculomotor nerve palsy (ONP) is often discovered in the ophthalmology department, manifested as ptosis with the same side, eyeball in the fixed external booth, or accompanied by limited inward, upward, and downward movements. The present case report described the effect of electroacupuncture (EA) on a breast cancer patient with ONP after chemotherapy.

**Patient concerns:**

A 56-year-old breast cancer patient presented with severe ptosis and fixed right eye exotropia. Besides, it is challenging to perform the movement inward, upward, and downward, and with obvious diplopia.

**Diagnoses:**

The breast cancer patient was diagnosed with ONP, chemotherapy history.

**Interventions:**

The patient was introduced to acupuncture department to receiving EA treatment.

**Outcomes:**

After 12 times of EA treatments, the symptom of ptosis was significantly improved, and the right upper eyelid can lift autonomously as same as the left eye. Besides, the patient’s right lateral eye could move freely, and the symptoms of double vision disappeared.

**Conclusion:**

The case suggests that EA may be an effective alternative treatment for ONP.

## 1. Introduction

Oculomotor nerve paralysis (ONP) is a common disease discovered in the ophthalmology department, mainly manifested as ptosis with the same side, eyeball in the fixed external exhibition space, the movement of inward, upward, and downward are limited, which can lead to some complications such as exotropia, ptosis, diplopia, and others.^[1,2]^ The etiology of ONP is complex and diverse, and microcirculation disorder is considered to be the leading cause.^[3,4]^ In various publications, some researchers have established the fact that acupuncture therapy is a safe and feasible method for ONP.^[5,6]^ However, the effect of electroacupuncture (EA) for breast cancer patients with ONP after chemotherapy was rarely reported. Here, we described a case of a 56-year-old breast cancer patient with ONP, who was successfully treated by using EA therapy as choosing the acupoints of Jingming (BL1), Cuanzhu (BL2), Yuyao (EX-HN4), Sizhukong (TE23), Tongziliao (GB1), Taiyang (EX-HN5), Chengqi (ST1) on the lesion side.

## 2. Case presentation

### 2.1. Clinical presentation

A 56-year-old woman with breast cancer was admitted to the Department of Breast Medical Oncology of Zhejiang Cancer Hospital to seek continued chemotherapy for further antitumor treatment. After receiving the second chemotherapy (nab-paclitaxel), she appeared to the symptoms of visual double shadow and the ptosis on the right side began to appear and deteriorated gradually. The neurologist diagnosed as ONP and considered the correlation between the disease of breast cancer and chemotherapy, the patient was treated with nutritional neuro drugs (Mecobalamin tablets), but the therapeutic effect was poor. To treat her eye disease, she was introduced to the traditional Chinese medicine department for acupuncture treatment.

We reviewed the patient’s previous medical history. She was diagnosed with breast cancer and received surgery and 8 cycles of chemotherapy treatment (liposomal adriamycin + cyclophosphamide for 4 cycles, and docetaxel for 4 cycles) 5 years ago. The recent PET-CT scan showed that her breast cancer relapsed and the cancer metastasized in bilateral supraclavicular and right parasternal lymph nodes, and multiple metastases occurred in the liver, sternum, T6, L5 vertebral body, sacrum, and left acetabular bone. As appeared the symptoms of ONP, the breast oncologist performed a cranial MRI on her, and the results were normal. D-dimer was 427 ng/mL by checking the coagulation function.

### 2.2. Diagnosis assessment

The patient had severe ptosis of the right eye, which covered the eyeball completely. Using external force to open the right side of the upper eyelid, it was seen that the eyeball was fixed exotropia, and the movement inward, upward, and downward could not be completed. In addition, bilateral pupils are equal in size, sensitive to light reflection, have no swelling and tremor of the binocular ball, 10 mm left eye crack, 0 mm right eye crack, and obvious diplopia of the visual object with inaccurate finger positioning, but the vision did not weaken and with no facial nerve function problem, and the muscle strength and tension of limbs were normal, neostigmine test negative. In the past year, the patient complained of fatigue and difficulty in relieving after rest. She had inadequate sleep at night, mainly demonstrated by difficulty falling asleep, suffering from many dreams, and waking up quickly. In addition, she felt numbness in her hands and feet after chemotherapy, especially in toes. According to the above clinical manifestations, she was diagnosed with ONP, breast cancer with multiple metastasis, chemotherapy-induced peripheral neuropathy, insomnia, and fatigue.

## 3. Intervention and outcome

**Figure 1.**
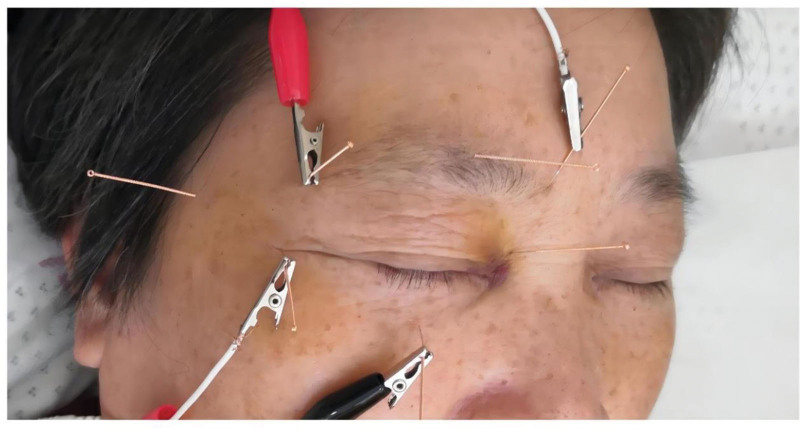
The selection of acupoints and EA treatment. The acupoints of BL1, BL2, EX-HN4, 23, GB1, EX-HN5, and ST1 on the right side were selected. BL2 and TE23, GB1, and ST1 were received EA treatment. EA = electroacupuncture.

EA therapy was performed to relieve her symptoms of ONP. The acupoints were chosen as BL1, BL2, EX-HN4, TE23, GB1, EX-HN5, and ST1 on the right side. The location of these acupoints (shown in Table 1) followed the WHO standard.**^[7]^** The disposable acupuncture needles of 0.18 mm × 25 mm type were selected. The patient was in the horizontal position, and the acupoints area was sterilized. When acupuncturing BL1, her eyes should close. The acupuncturist used his left hand to gently push the eyeball toward the lateral side and fix it and used his right hand to slowly insert the needle perpendicularly about 20 mm deep inside along the orbital margin, twirled or lifted and thrust the needle slightly, to make the patient feel eyeball with acid expansion feelings. The acupuncture needle direction toward BL2 to BL1, EX-HN4 penetrated through TE23, TE23, and GB1 were stabbed to the direction of EX-HN5 along the temporal side, and the EX-HN5 was stabbed sideways to the direction of pupil. These 6 points were all punctured inside about 18 to 23 mm depth, to make the right brow ridge and frontotemporal area of the patient obviously with the feeling of acid bilge. When acupuncture ST1, the eyeball was pushed up, and the needle was stabbed inside about 18 mm depth along the infraorbital margin. BL2 and TE23, GB1and ST1 were connected with EA treatment by using HuaTuo SDZ-IIB acupoint neural stimulator. The EA frequency was 2Hz and the intensity was comfortable to the patient. The EA treatment was performed 30 minutes each time and 3 times per week (every other day). The selection of acupoints and EA treatment was shown in Figure 1.

**Table 1:** Location of acupoints.

Acupoints	Location
Jingming (BL1, right side)	In the depression superior to the inner canthus
Cuanzhu (BL2, right side)	In the depression on the medial eye of eyebrow, on the supraorbital notch
Yuyao (EX-HN4, right side)	In the middle of the eyebrow. When one is looking straight forward, the point is directly above the pupil
Sizhukong (TE23, right side)	In the depression at the lateral end of eyebrow
Tongziliao (GB1, right side)	0.5 cun lateral to the outer canthus on the lateral side of the orbit
Taiyang (EX-HN5, right side)	In the region of the temples, in the depression about 1 finger-breadth posterior to the midpoint between the lateral end of the eyebrow and the out canthus
Chengqi (ST1, right side)	With the eyes looking straight forward, the point is directly below the pupil of the eye, between the eyeball and the infraorbital ridge

**Figure 2.**
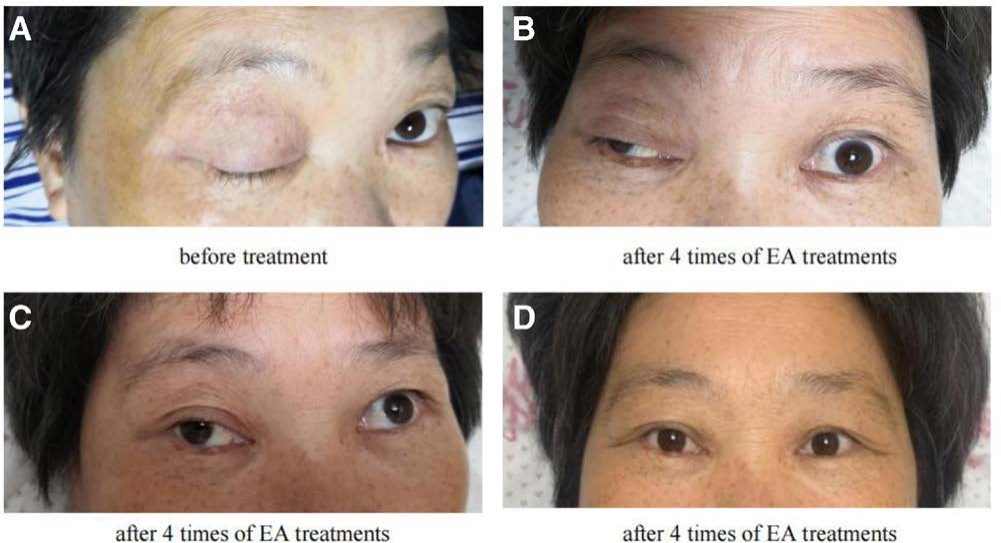
The changes of ONP symptoms during the EA treatment process. (A) Before treatment. (B) After 4 times of EA treatments. (C) After 8 times of EA treatment. (D) After 12 times of EA treatment. EA = electroacupuncture, ONP = oculomotor nerve paralysis.

Before the first treatment, the upper eyelid of the patient’s right eye could not be lifted independently; the right eye was 0 mm cracked, the right exotropia was fixed, and diplopia (shown in Fig. 2A). After 4 times of EA treatments, the upper eyelid of the patient’s right eye could lift slightly, with the eye crack of about 4 mm, but the right exotropia was still fixed and diplopia (shown in Fig. 2B). After 8 times of EA treatments, the upper eyelid of the patient’s right eye could lift up obviously, the eye crack widened to 7 mm, and the right eyeball could move slightly outwards. The patient said the symptoms of diplopia were relieved but still remained (shown in Fig. 2C). After 12 times of EA treatments, the upper eyelid of the patient’s right eye could be lifted autonomously, and the eye crack increased to about 9 mm, which was basically the same as the left eye. The right eye could move up and down autonomously, with slight adduction and the symptom of diplopia almost disappeared (shown in Fig. 2D). During the periods of EA treatment, the patient completed 2 cycles of chemotherapy treatment smoothly. In the following 2 months, the patient’s symptom of ONP was no recurrence and the next stage of chemotherapy was also carried out smoothly.

## 4. Discussion

The oculomotor nerve mainly dominates the muscles around the eye, including the levator palpebrae superioris, rectus superior, rectus inferior, rectus internus, and obliquus inferioris.^[2]^ It often manifests as ptosis of the same side of the upper eyelid, eyeball in the fixed external booth, inward, upward, and downward movements are limited, which could cause some complications, such as exotropia, ptosis of the upper eyelid, diplopia, etc. Current studies show that,^[1]^ ONP is usually caused by the pathological changes of the oculomotor nerve itself or its surrounding tissue, but the etiology can be complex and diverse, which may be related to some inflammatory diseases, angiopathy, intracranial lesions, craniocerebral trauma, cancer, diabetes, and other factors.^[8,9]^ For example, long-term hyperglycemia could cause microvascular disease, ischemia, anoxia, abnormal energy metabolism of nerve transmission, oculomotor nerve blood supply, and energy metabolism affected to cause ONP to appear.^[10,11]^ Intracranial aneurysm, the intracranial space-occupying lesion, or cancers that metastasize and invade the central nervous system in the Intracranial may lead to ONP. Besides, it may be cancer metastasis directly invades any part of the oculomotor nerve or adjacent tissues, which will also result in oculomotor nerve paralysis. However, neither of these cases is suitable for the patient in this case report. Changes in the body microenvironment and microcirculation disturbance of this patient, which were caused by multiple cancer metastasis may be the underlying factors leading to ONP. A study^[12]^ pointed out that the peripheral blood of patients with cancer is often in a hypercoagulable state which creates conditions for cancer growth and metastasis, but at the same time, it will lead to the anoxic state of microcirculation and some lesions of the body. The patient had multiple cancer metastases, and her D-dimer is much higher than normal, which can be considered that there is a hypercoagulable state and poor metabolic capacity of microcirculation in her body. Other studies^[3,4]^ suggest that the main cause of ONP is local microcirculation disorder, which leads to the abnormal energy metabolism of the oculomotor nerve.

Besides the effects of cancer and its metastasis, the chemotherapy treatment may also be a potential pathogenic factor. Peripheral neurotoxicity is a common side effect of many chemotherapeutic drugs, especially in chemotherapeutic drugs of platinum, taxanes, and vinblastine.^[13,14]^ However, peripheral neuropathy caused by chemotherapeutic drugs is mostly characterized by the end of limb numbness, sensory loss or paresthesia (such as tingling sensation, electric shock sensation, ant crawling sensation, foreign body sensation, etc), decreased tendon reflex, sensory ataxia, which can be triggered or aggravated in case of cold stimulation.^[15]^ Although the probability is low, the toxicity of chemotherapy drugs may also lead to ONP by invading peripheral oculomotor neuropathy. It is generally believed that chemotherapeutic drugs usually do not pass through the blood–brain barrier and have little effect on the central nervous system. While in recent years, some researchers have put forward the concepts of “chemotherapy brain or “chemotherapy fog.”^[16–18]^ It means that chemotherapy drugs can cause cognitive impairment and hypomnesis.^[19,20]^ Therefore, it can be considered that the side effects of chemotherapy drugs may be a risk factor for central nervous system diseases and the possibility of affecting the cranial nerve.

The literature review showed that acupuncture had a definite curative effect on oculomotor nerve paralysis, and Jingming (BL1), Cuanzhu (BL2), Yangbai(GB14), Taiyang (EX-HN5), Yuyao (EX-HN4), Sizhukong (TE23), Sibai(ST2), Chengqi (ST1), Tongziliao (GB1) were the most widely used acupionts.^[21]^ Some studies have pointed out that^[22,23]^ acupuncture at the local points of the orbit could stimulate the muscles, which dominate the movement of the eyeball in orbit, improving muscle activity and relieving paralysis symptoms. For example, acupuncture at BL1 could stimulate the internal rectus muscle in the eye directly, and acupuncture at ST1 point could stimulate the inferior rectus muscle and inferior oblique muscle under the eyeball.^[21]^ Furthermore, acupuncture at the local points of the orbit could regulate the circulation of peripheral micro blood vessels and improve blood flow, the flow rate of capillaries and the energy metabolism would be promoted so as to accelerate the recovery of orbital muscle and nerve function. When acupuncture acupoints around the orbit, the acupuncturist should be bold and careful, and the direction, depth, and intensity of the acupuncture should be well controlled. BL2 and TE23, GB1, and ST1 acupoints were connected with 2Hz EA, and the intensity should be appropriate for the patient’s slight sense of tears so as to achieve enough stimulation. After the EA treatment, not only ONP symptoms of the patient were completely relieved, but the sleep quality and fatigue state were significantly improved as well. She said her whole mood became much more cheerful. During the EA treatment, the patient successfully completed 2 times of chemotherapy treatments without significant side effects.

## 5. Conclusion

This case report is innovative because it shows that EA is effective for treating ONP in breast cancer patients after chemotherapy. Due to the limitation of the individual case, large randomized clinical trials with adequate observation and follow-up are warranted to demonstrate the curative effects of EA.

## Author contributions

**Writing – original draft:** Chun Du.

**Funding acquisition:** Chao Lu.

**Investigation:** Chao Lu.

**Resources:** Chao Lu, Guangliang Li.

**Writing – review & editing:** Chao Lu, Dehou Deng.

**Supervision:** Weiji Chen.

**Validation:** Weiji Chen.
